# Observation for the effect of rTMS combined with magnetic stimulation at Neiguan (PC6) and Sanyinjiao (SP6) points on limb function after stroke: A study protocol

**DOI:** 10.1097/MD.0000000000022207

**Published:** 2020-09-18

**Authors:** Penglong Yu, Yuan Wang, Jie Yuan, Jie Chen, Yaling Lei, Zucheng Han, Dongling Liu, Yuan Zhao, Pei Wang, Fan Luo

**Affiliations:** a Hospital of Chengdu University of Traditional Chinese Medicine, Chengdu, Sichuan Province; b Department of Encephalopathy, Shaanxi Provincial Hospital of Traditional Chinese Medicine; c Insomnia Research Center of Shanxi Administration of Traditional Chinese Medicine, Xi’an; d School of Clinical Medical, Shannxi University of Chinese Medicine, Xianyang; e School of Basic Medical Sciences, Chengdu University of Traditional Chinese Medicine, Chengdu, China.

**Keywords:** clinical trial, limb dysfunction, PC6, research protocol, rMS, rTMS, SP6, stroke

## Abstract

**Background:**

Stroke is the primary cause of adult disability in China, which causes serious personal, family, and social burden. “Central peripheral central” closed-loop rehabilitation theory is proved to be an effective neural rehabilitation model. Based on this theory, repetitive transcranial magnetic stimulation (rTMS) combined with magnetic stimulation of Neiguan (PC6) and Sanyinjiao (SP6) may be an effective treatment for limb dysfunction after stroke. However, the efficacy and mechanism of repetitive magnetic stimulation of M1 region combined with magnetic stimulation of Neiguan and Sanyinjiao points on limb dysfunction after stroke has not been confirmed.

**Methods/Design:**

This study is a prospective, randomized, controlled, open trial. We randomly divided 42 subjects, aged 35 to 80 years, diagnosed with ischemic stroke within 1 month, into 2 groups with a ratio of 1:1. On the basis of this medical treatment, patients in the experimental group received 1 Hz rTMS in M1 area on the contralateral side, and 3 Hz rTMS treatment at Neiguan point and Sanyinjiao point on the affected side. The control group was treated with acupuncture (body acupuncture). All patients were treated once a day and followed up for 10 days. The National Institute of Health Stroke Scale score, simplified fulg Meyer, modified Barthel index, and cortical excitability were evaluated on the day of enrollment and the 10th day of treatment respectively. The modified Barthe index was followed up on the 30th day of treatment, and the adverse reactions were recorded at any time. The mechanism of rTMS will be revealed by Barthe index before treatment, on the 10th day of treatment and on the 30th day of follow-up. The results were analyzed by spss19.0 software, and the quantitative indexes were analyzed by *t* test and rank sum test. *χ*^2^ test was used for non-grade counting, and rank sum test was used for grade counting. All statistical tests were performed with bilateral test. If *P* value is less than or equal to .05, the difference will be considered statistically significant.

**Conclusion:**

The purpose of this study was to determine the effect of repetitive magnetic stimulation of M1 region combined with magnetic stimulation of Neiguan and Sanyinjiao points on limb function after stroke. Through this study, we expect to explore a new scheme for the treatment of poststroke dyskinesia, and prove that compared with rTMS and acupuncture alone, the closed-loop rehabilitation theory based on “center peripheral center” can be more efficient and safe in the treatment of poststroke limb dysfunction.

**Trial Registration:**

The trial was registered in China clinical trial registry (http://www.chictr.org.cn/index.aspx), ID: ChiCTR1900026890 (October 25, 2019)

## 1 Introduction

Stroke is one of the main diseases that lead to human death and disability. According to statistics, about 1/7 of women and 1/10 of men in Europe die directly or indirectly from stroke every year.^[1]^ In the United States, 800,000 people suffer from stroke every year. ^[2]^ Stroke is the main disease leading to human disability. About 2/3 of stroke patients have limb dysfunction,^[3]^ which seriously affects people's quality of life and physical and mental health, and brings huge economic and psychological burden to families and society.

Nerve rehabilitation is the main measure to restore limb dysfunction in stroke patients. The traditional rehabilitation treatment mainly includes peripheral intervention measures, such as traditional occupational therapy, physical therapy, acupuncture treatment, etc., all of which are through the continuous input of stimulation from the sensorimotor system to the central nervous system, strengthening the training of the correct movement mode to correct the abnormal movement mode, so as to promote nerve recovery. In particular, acupuncture therapy has been widely used in the rehabilitation treatment of stroke in recent years.^[4]^ However, due to the differences in acupuncture schools, acupuncture methods and acupoint selection, and the stimulation intensity cannot be quantified, the heterogeneity of experimental results is relatively large,^[5]^ and it is difficult to obtain reliable clinical research data.

With the development of rehabilitation technology, through a variety of precise positioning, noninvasive direct brain stimulation in the injured or functional brain areas is gradually emerging. rMS,rTMS,brain-computer interface, mirror therapy, etc. Compared with traditional acupuncture therapy, transcranial magnetic stimulation and peripheral acupoint magnetic therapy can not only play the same therapeutic effect as traditional acupuncture therapy, but also have the advantages of no radiation, no trauma, and quantitative observation of stimulation intensity.^[6]^

To combine the advantages of peripheral and central interventions, a new neural rehabilitation model called “central peripheral central” closed-loop rehabilitation theory was proposed. According to the theory, “peripheral intervention” can superimpose the therapeutic effects of the 2 when the “central intervention” makes the brain area activated, and become a rehabilitation treatment mode with expectant outcomes.^[7]^ Based on this theoretical model, we have worked out a treatment plan of repetitive transcranial magnetic stimulation (rTMS) at Neiguan (PC6) and Sanyinjiao (SP6) for limb dysfunction after stroke.

rTMS is an effective central stimulation for poststroke dyskinesia confirmed by evidence-based medicine.^[8]^ As a noninvasive stimulation technique, rTMS can detect the changes of motor-evoked potential (MEP), electroencephalogram, cerebral blood flow, metabolism, and brain function from the changes of neural activity, especially low-frequency (1 Hz) rTMS can effectively stimulate the central nervous system noninvasively. As a motor center, M1 area of cerebral cortex plays an important role in our limb movement, and has been an effective stimulation target for the treatment of limb dysfunction after stroke.

PC6 and SP6 are the main points of “Xingnao Kaiqiao” acupuncture method in the treatment of stroke, and they are also high-frequency acupoints in the relevant literature on the treatment of stroke.^[9]^ It is found that acupuncture at PC6 in patients with acute cerebral infarction can activate frontal lobe, parietal lobe, and motor area.^[10,11]^ Acupuncture at SP6 in healthy people can activate specific bilateral brain areas such as frontal, parietal, temporal cortex, cingulate gyrus, and thalamus.^[12]^ However, magnetic stimulation of PC6 and SP6 can not only activate brain regions, but also increase the link between brain regions, and strengthen the information exchange between brain regions.^[13]^ This shows that the effect of acupoint magnetic stimulation is similar to that of acupuncture, and compared with acupuncture, magnetic stimulation is noninvasive and pain free, and can improve the compliance of patients.

However, it is a pity that the mechanism of acupoint magnetic therapy in the treatment of poststroke limb dysfunction is still unclear, and the clinical research is relatively insufficient. To clarify the effect of repetitive transcranial magnetic stimulation M1 combined with repetitive magnetic stimulation at PC6 and SP6 on limb function after cerebral infarction, a prospective randomized, controlled, and open trial was conducted. Forty-two patients with ischemic stroke within 1 month after onset were randomly divided into acupuncture group (control group) and repetitive magnetic stimulation group (experimental group). The purpose of this study is to explore a new method to promote the recovery of limb function after stroke.

## 2 Methods/design

### 2.1 Study design

This study is a single-center, randomized, controlled, open clinical trial. A total of 42 participants will be randomly divided into 2 groups with a ratio of 1:1. Both groups were given basic treatment (cerebrovascular disease medical treatment, rehabilitation treatment). In addition to the basic treatment, the experimental group received 1 Hz rTMS in M1 area of the healthy side, and 3 Hz rMS treatment at PC6 and SP6 on the affected side. The control group was treated with acupuncture.

**Figure 1:**
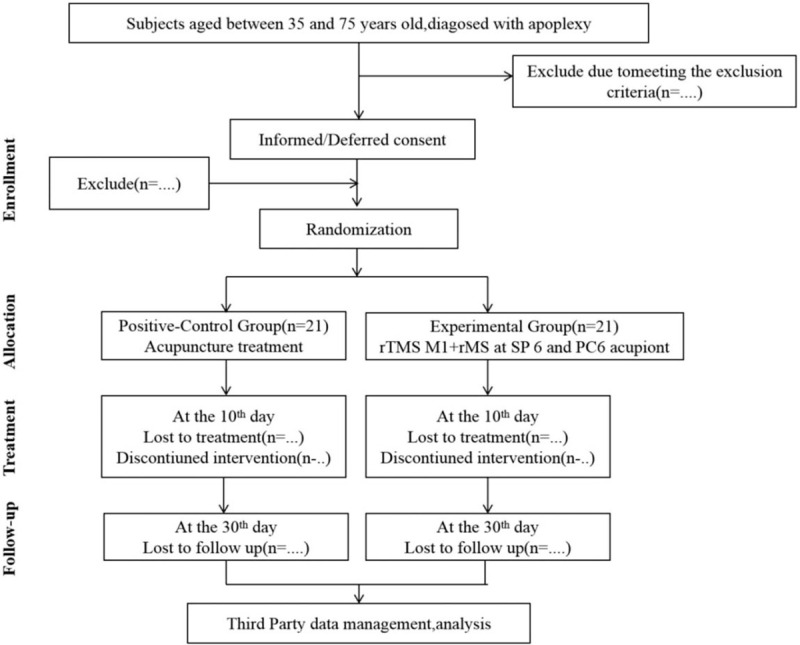
Flow of subjects randomized to receive acupuncture treatment or rTMS at M1 +rMS at SP6 and PC6 acupiont.

The whole study period was 30 days, including 10 days of treatment and 20 days of follow-up. Before treatment and on the 10th day of treatment and the 20th day of follow-up, the clinical efficacy evaluation scale was used to evaluate the subjects, and the cortical excitability was detected by rMS before and on the 10th day of treatment (Fig. 1).

The clinical trial results will be reported according to the Standards for Reporting

Interventions in Clinical Trials of Acupuncture (STRICTA) statement.^[14]^

### 2.2 Ethics

This study has passed the ethics committee Shaanxi Provincial Hospital of traditional Chinese medicine ((2019)(32)), and registered on October 5, 2019 at http://www.chictr.org.cn/index.aspx, ID is chictr19000026890. Informed consent has included all the subjects involved. Any modification of the study protocol or informed consent, which may affect the rights and interests of the participants or the implementation of the study, should be reported to the Ethics Committee for approval again. If there are any serious adverse events in the trial, the ethics committee should review it in time and put forward written suggestions for modification, including sufficient power to suspend the trial.

### 2.3 Study population

#### 2.3.1 Inclusion criteria

1. One month after onset, the patients were diagnosed as ischemic stroke according to computed tomography/magnetic resonance imaging (CT/MRI) examination.
2. Aged 35 to 75 years.
3. Infarction focus was basal ganglia or (and) radiation coronal area, with unilateral limb paralysis (4 <= National Institute of Health Stroke Scale (NIHSS) <= 15).
4. Be conscious and voluntary to join the experiment and sign the informed consent.

#### 2.3.2 Exclusion criteria

1. Past history of stroke with limb paralysis.
2. Ischemic stroke caused by cardiogenic, other, and unknown causes.
3. Patients with severe chronic diseases such as heart, liver, kidney, and hematopoietic system affected the treatment.
4. Past history of mental retardation, mental disorder, and epilepsy.
5. Indications for thrombolysis.
6. Systolic blood pressure (> = 180 mm Hg) or diastolic blood pressure (> = 120 mm Hg).
7. The space-occupying effect is obvious, with CT or MRI signs of midline structural displacement.
8. Metal foreign bodies exist within 30 cm of the treatment site, such as cochlear implant, built-in pulse generator, aneurysm clamp, stent, metal internal fixation, etc.
9. History of alcohol abuse or long-term use of cortical stimulants (sedatives and antidepressants) before illness.

#### 2.3.3 Withdrawal criteria

1. After inclusion, it was found that they were not suitable to continue to participate in the study.
2. Those who fail to treat according to the regulations affect the evaluation of curative effect.
3. Withdrawal of consent by the subject or a legal representative.
4. Loss to follow-up.
5. Serious adverse events.
6. Significant errors or deviations were found in the clinical protocol.
7. New infarct or secondary hemorrhage occurred and the condition deteriorated.

### 2.4 Study settings and recruitments

The present study will be conducted in the Shaanxi Province Hospital of Traditional Chinese Medicine.

This study intends to recruit patients diagnosed with acute ischemic stroke within 1 month in the outpatient and inpatient departments of Shaanxi Provincial Hospital of traditional Chinese medicine. At the same time, posters, leaflets, and publicity should be put up in a prominent position in the hospital, so that patients can have regular free examination, which will help to successfully complete the recruitment work. At the same time, the official website, wechat, and microblog of Shaanxi Provincial Hospital of traditional Chinese medicine are powerful publicity methods.

### 2.5 Study group

In this study, 42 patients who met the inclusion criteria and obtained informed consent were selected as the research subjects. The subjects were randomly divided into 2 groups according to the ratio of 1:1: the experimental group and the control group, with 21 cases in each group.

### 2.6 Study time

This clinical study will be conducted from August 1, 2019 to August 1, 2021.

### 2.7 Interventions

The intervention measures involved in this study are based on the theory and professional practice knowledge of traditional Chinese medicine. The doctors who are in charge of operating the instruments for treatment have obtained the practicing certificate issued by the national health and Health Commission, and have received more than 3 years of clinical skills training in acupuncture and rTMS. Patients in the experimental group were treated with rMS in M1 area of healthy side, PC6, and SP6 on the affected side, while patients in the control group received acupuncture treatment. In addition, both groups were given basic treatment.

#### 2.7.1 Experimental group

**Figure 2:**
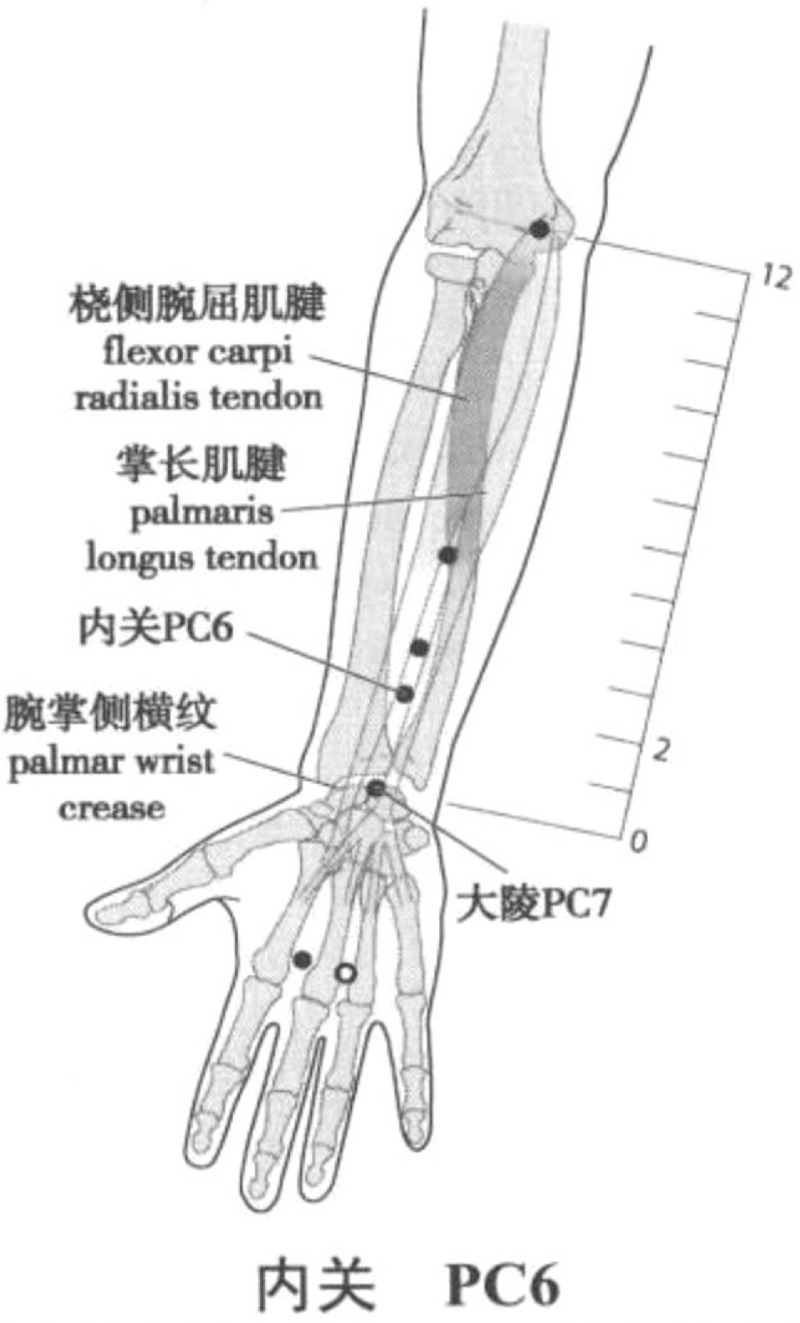
Location of Neiguan (PC 6) Acupoint. Longxiang H. WHO Standard Acupuncture Point Locations in the Western Pacific Region (Chinese-English bilingual edition) [M]. Beijing: People's Medical Publishing House, 2010.

**Figure 3:**
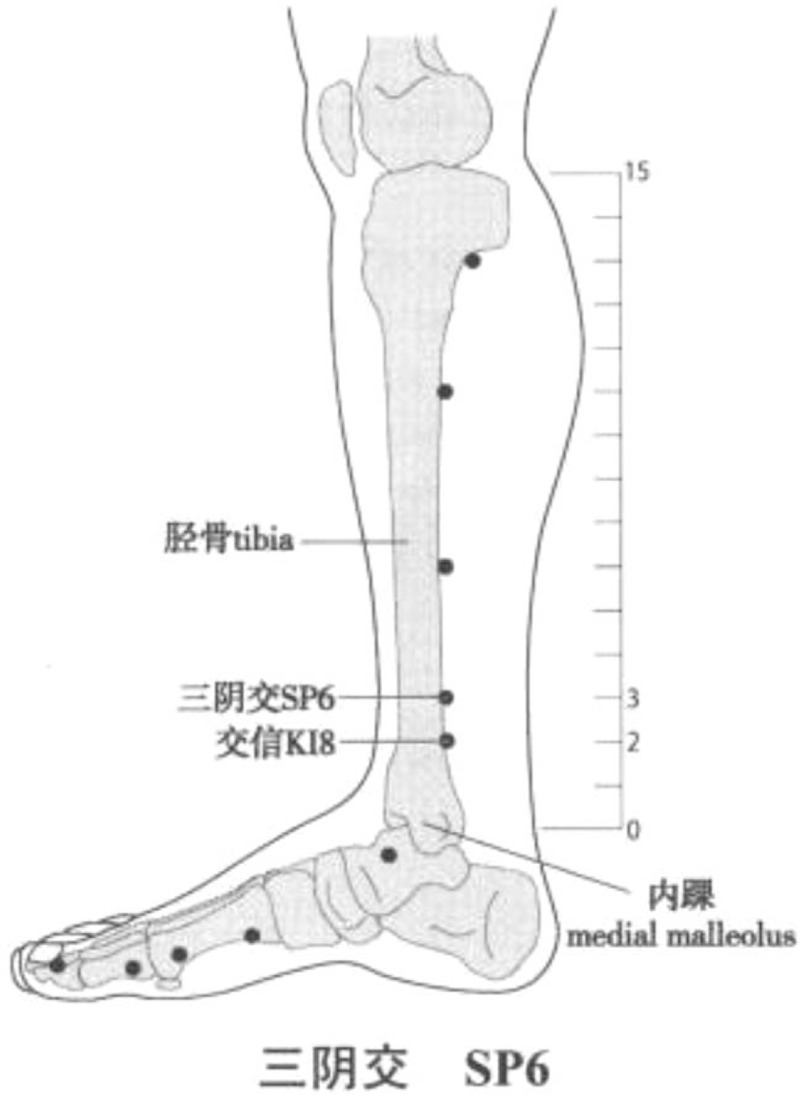
Location of Sanyinjiao (SP 6) Acupoint. Longxiang H. WHO Standard Acupuncture Point Locations in the Western Pacific Region (Chinese-English bilingual edition) [M]. Beijing: People's Medical Publishing House, 2010.

rTMS therapeutic instrument (ccy-ii, Wuhan yiruide medical products New Technology Co, Ltd, Wuhan) was used. According to the theory of modern neural rehabilitation,^[15,16]^ rTMS stimulation of contralateral M1 area was applied to the rehabilitation of limb function after stroke. According to the Xingnao Kaiqiao acupuncture method created by Professor Shi Xuemin, academician of Chinese Academy of engineering and master of Chinese medicine,^[17,18]^ PC6 and SP6 on the affected side were selected as the operation sites. After rest preparation, the transcranial magnetic stimulation technology and resting motor threshold (RMT) were tested: the patients took standard sitting posture, maintained emotional stability, and muscle relaxation. When stimulating M1 area, the coil should be tangent to the scalp. Adjust the coil to obtain the maximum amplitude and the best MEP repeatability. The stimulation intensity will start from 30% and increase gradually at the rate of 2.5%. The minimum stimulus intensity (RMT) was 5 abdominal contractions of the contralateral abductor brevis muscle in 10 consecutive stimuli; localizing the stimulation area: when stimulating the motor cortex of the thumb on the right dorsolateral prefrontal cortex (r-dlpfc) on the scalp, the stimulation coil was placed on the right dorsolateral prefrontal cortex (r-dlpfc) on the scalp, then 6 cm to the right; during peripheral stimulation, the stimulation coil will be placed on the affected side of PC6 (located on the palmar side of the forearm, on the line between Quze acupoint and DalingQuze acupoint, 2 inches on the transverse line of the wrist, between the tendon of palmaris longus and the flexor carpi radialis) (Fig. 2). The stimulation coil will be placed at the SP6 on the affected side (3 inches above the tip of the medial malleolus, and the posterior edge of the tibia is close to the bone edge depression) (Fig. 3); setting stimulation parameters: according to the different stimulation sites, different standard stimulation intensity modes were selected: the frequency was 1 Hz, the total number of pulses was 900, and the intensity of RMT was 90%; in the peripheral PC6 and SP6 position, the frequency was 3 Hz, the total number of pulses was 450, and the intensity of RMT was 90%. After checking the above parameters, the stimulation was started and the loop valve was removed. Participants will be treated once a day for 25 minutes for 10 consecutive days.

#### 2.7.2 Positive-control group

In addition to the basic treatment, the patients in the control group also need to be given corresponding acupuncture treatment. According to the theory of traditional Chinese medicine, acupuncture position and parameters were selected^[19,20]^: upper limbs (bilateral): Hegu, Neiguan, Shousanli, Quchi, Jianli; lower limbs (bilateral): Taichong, Xuanzhong, Sanyinjiao, Zusanli, Yanglingquan, Fengshi; head: Baihui, Fengchi (bilateral). Patients will be treated once a day for 10 consecutive days.

### 2.8 Adverse events observation

#### 2.8.1 Adverse reactions and countermeasures of magnetic stimulation

Magnetic stimulation may induce epilepsy, local skin numbness, pain and discomfort, and other adverse reactions. In the treatment process, the intensity, frequency, and stimulation times can be changed according to the patient's condition to reduce the occurrence of adverse reactions.^[21–23]^ During the study, a patient treatment log will be established to monitor all adverse reactions during treatment and follow-up, and appropriate intervention will be carried out. If serious adverse reactions occur during the treatment, they should be reported to the principal investigator immediately, and the details should be recorded in the patient's treatment log and corresponding treatment measures should be taken, such as adjusting the coil position and stimulation intensity to avoid stimulating the sensitive orbitofrontal cortex (100% RMT → 80% RMT), or giving appropriate medical intervention according to the patient's condition. If the subject or researcher thinks that the patient cannot tolerate these adverse reactions, the trial can be terminated and the subject will withdraw from the study.

#### 2.8.2 Adverse reactions of acupuncture and countermeasures

Acupuncture may cause acupuncture syncope, convulsion, severe pain, stuck needle, broken needle, local infection and subcutaneous hematoma and other adverse reactions. However, they are generally mild reactions, and the prognosis is generally good after corresponding treatment.^[24]^ According to the causes of adverse reactions and the degree of adverse reactions, symptomatic treatment can be carried out, such as: detailed medical history to avoid convulsion, appropriate psychological counseling to avoid fainting, standardized needle application, auxiliary manipulation to avoid needle sticking, pain, broken needle, etc. If there are serious adverse reactions, they should be reported to the principal investigator immediately, and the details of adverse reactions should be recorded in the patient's log, and medical intervention should be carried out. If the subject or researcher thinks that the patient cannot tolerate these adverse reactions, the trial can be terminated and the subject will withdraw from the study.

### 2.9 Primary outcomes

NIHSS, score, and simplified fulg Meyer (FMA) score were evaluated before treatment and on day 10. The modified Barthel Index (MBI) was evaluated before and 10 days after treatment and 30 days after treatment

### 2.10 Secondary outcomes

Cortical excitability was evaluated on the day of admission and the 10th day of treatment. The patients’ mood, sleep, and diet were followed up through the treatment log. All adverse reactions and causes of shedding will be recorded in case report form (CRF) in time.

### 2.11 Estimation of sample size

This study is a preliminary clinical study to evaluate the efficacy and mechanism of targeted therapy before, after and during the follow-up period. We plan to enroll 42 participants in this study. (In this study, NIHSS score was used as the effect value. According to the sample size formula of good and bad clinical trials: N = 2δ^2^ × f(α,β)/(u_1_−u_2_)^2^. In the acupuncture group, the NIHSS mean value before treatment was 8.95, the standard deviation was 1.93, and the average NIHSS after treatment was 6.90, sou_1_ = 8.95, u_2_ = 6.90, δ = 1.93, α = 0.05, β = 0.1, f (α, β) = 10.5 from the table, which was substituted into the formula to calculate the sample size n ≈ 19 cases. Considering the loss rate of 10%, the total sample size required was 19 × 2 × (1 + 10%) ≈ 42 cases.)

### 2.12 Randomization

After clinical screening and signing the informed consent form, 42 subjects were randomly assigned to the control group and the experimental group according to the proportion of 1:1. This study is an open trial, without blinding, using third-party evaluation and statistics. Each subject will receive different individualized forms of stimulation, while all subjects are aware of the grouping.

### 2.13 Statistical analysis

According to the situation of data collection, data analysis will be based on the principle of intention to treat analysis (ITT) and/or per protocol analysis (PP). We will also evaluate the group effect by comparing the analysis results of the above 2 data sets to evaluate the accuracy of our analysis results.

All data will be analyzed by third-party statisticians using spss23.0 (v23.0, SPSS Inc, Chicago, IL). *P* < .05 was significant. Descriptive statistics will be carried out by group and time for average, standard deviation, maximum value, minimum value, and so on.

The data of the patients were collected and summarized. The quantitative indexes were *t* test and rank sum test. *χ*^2^ test (ANOVA) was used for non-rank counting, and rank sum test was used for grade counting. All statistical tests were performed with bilateral test. If *P* value is less than or equal to .05, the difference will be considered statistically significant.

#### 2.13.1 Primary outcomes

NIHSS score and simplified fulg Meyer score were evaluated before and 10 days after treatment, and the data were analyzed by rank sum test or *χ*^2^ test. The modified Barthel index was evaluated before and 10 days after treatment and 30 days after treatment. The data were analyzed by rank sum test or *χ*^2^ test. The statistical significance between groups will show that there are differences in modified Barthel index, NIHSS score, and simplified fulg Meyer among groups after intervention.

#### 2.13.2 Secondary outcomes

The cortical excitability was evaluated on the day of admission and the 10th day of treatment respectively. The data obtained from the evaluation were analyzed by one-way ANOVA, and the classified result variables in the treatment log were compared by *χ*^2^ test.

#### 2.13.3 Safety evaluation

Adverse events recorded in the patient log will be analyzed as multiple variables. The number and percentage of AE patients were calculated and compared by *χ*^2^ test.

### 2.14 Quality control

During the whole treatment and follow-up process, patients’ withdrawal and the reasons for withdrawal will be recorded in time. In order to ensure the test quality, the quality supervisor will regularly check all process details and check the authenticity of the data. In addition, the scientific research department of Shaanxi Provincial Hospital of traditional Chinese medicine will be invited to manage the data independently.

Since differences between physicians may lead to bias, each evaluation of the scale will be performed by a designated physician trained in the same evaluation criteria. The 2 physicians in charge of the treatment will receive specialized training in research and operation, including the use of transcranial magnetic stimulation and acupoint localization, prior to recruitment. In order to record treatment compliance, we made a record card for patients, including treatment date, personal information, and signature after each treatment.

## 3 Discussion

Limb dysfunction after stroke is the main complication of stroke and the main cause of adult disability, which seriously affects the working ability and quality of life of stroke patients, and brings serious economic burden to patients and society. With the progress of medical technology, patients have higher expectations of limb function recovery after stroke. Therefore, we designed the clinical research program, hoping to obtain high-quality research data through the improvement of patients’ symptoms and the scale, so as to provide reliable basis for future multicenter research. As a traditional Chinese rehabilitation therapy, acupuncture and moxibustion is widely recognized in the clinical treatment and rehabilitation of stroke. Therefore, we chose the acupuncture program with high public reliability as the control group. PC6 and SP6, as the experimental group, can activate the brain area, increase the link between the brain regions, and strengthen the information exchange between the brain regions. Compared with acupuncture and moxibustion, magnetic stimulation has the advantages of noninvasive, painless, and statistical. In previous clinical trials, it has been confirmed that low-frequency rTMS is of positive significance for the recovery of motor function of stroke patients.^[25]^ However, the use of acupoint magnetic stimulation in the treatment of limb motor dysfunction after stroke is often seen in case reports, and there is no systematic theoretical guidance. The closed-loop rehabilitation theory of “center peripheral center” has great enlightenment on how to improve the treatment effect of limb dysfunction after stroke, but there are few clinical curative effect observation with high confidence based on the “center peripheral center” closed-loop rehabilitation theory. Therefore, based on this theory, we plan to give magnetic stimulation combined with peripheral magnetic stimulation at PC6 and SP6 in central M1 area to observe the effect and difference of acupuncture group on limb function after stroke.

Our scheme is only a pilot study, and its shortcomings include: small sample size, no blindness, short observation time, etc. In the experiment, we will try our best to reduce the error which may affect the research results. Finally, we will prove that rTMS combined with magnetic stimulation of PC6 andSP6 is an effective treatment for limb dysfunction after stroke.

## Acknowledgments

This work was supported by Shaanxi Administration of Traditional Chinese Medicine, No. 2019-GJ-QT006

## Author contributions

Jie Yuan and Jie Chen designed this study together. Penglong Yu, Jie Yuan and Yuan Wang drafted the protocol. Jie Chen performed the statistical analysis. Yaling Lei was responsible for the writing revision. All authors read and approved the final manuscript.

**Acupuncture treatment:** Yuan Wang

**Data collection:** Yuan Wang, Fan Luo, Pei Wang

**Formal analysis:** Penglong Yu, Yuan Zhao.

**Funding acquisition:** Jie Chen, Jie Yuan

**Methodology:** Jie Yuan, Jie Chen

**Project administration:** Zucheng Han, Dongling Liu

**Project coordination:** Zucheng Han, Dongling Liu

**TMS treatment:** Yuan Zhao

**Writing – original draft:** Penglong Yu, Jie Yuan and Yuan Wang

**Writing – review & editing:** Yaling Lei, Jie Chen

## Abbreviations

Abbreviations: ANOVA = analysis of variance, CRF = case report form, FMA = Fulg-Meyer, ITT = intention-to-treat analysis, MBI = modify the Barthel index, MEP = motor-evoked potential, NIHSS = National Institute of Health Stroke Scale, PP = per-protocol analysis, rMS = repetitive magnetic stimulation, RMT = resting motor threshold, rTMS = repetitive transcranial magnetic stimulation, STRICTA = standards for reporting interventions in clinical trials of acupuncture.
